# H3.1 Eviction Marks Female Germline Precursors in *Arabidopsis*

**DOI:** 10.3390/plants9101322

**Published:** 2020-10-06

**Authors:** Elvira Hernandez-Lagana, Daphné Autran

**Affiliations:** DIADE, IRD, CIRAD, University of Montpellier, 911 avenue Agropolis, 34000 Montpellier, France; elvira.hernandez-lagana@ird.fr

**Keywords:** cell cycle, histone H3, reproductive competence, plasticity, ovule development, *Arabidopsis*

## Abstract

In flowering plants, germline precursors are differentiated from somatic cells. The female germline precursor of *Arabidopsis thaliana* is located in the internal (nucellar) tissue of the ovule, and is known as the Megaspore Mother Cell (MMC). MMC differentiation in *Arabidopsis* occurs when a cell in the subepidermal layer of the nucellar apex enters the meiotic program. Increasing evidence has demonstrated that MMC specification is a plastic process where the number and developmental outcome of MMCs are variable. During its differentiation, the MMC displays specific chromatin hallmarks that distinguish it from other cells within the primordium. To date, these signatures have been only analyzed at developmental stages where the MMC is morphologically conspicuous, and their role in reproductive fate acquisition remains to be elucidated. Here, we show that the histone 3 variant H3.1 HISTONE THREE RELATED 13 (HTR13) can be evicted in multiple subepidermal cells of the nucellus, but that H3.1 eviction persists only in the MMC. This pattern is established very early in ovule development and is reminiscent of the specific eviction of H3.1 that marks cell cycle exit in other somatic cell types, such as the root quiescent center (QC) of *Arabidopsis.* Our findings suggest that cell cycle progression in the subepidermal region of the ovule apex is modified very early in development and is associated with plasticity of reproductive fate acquisition.

## 1. Introduction

In *Arabidopsis thaliana*, female gamete formation occurs within the ovule in the adult organism. This process initiates with the specification of spore precursors from somatic cells that are located in the nucellus, in the subepidermal layer at the ovule primordium apex. Typically, a single female spore mother cell, called the Megaspore Mother Cell (MMC), adopts a reproductive fate and is committed to meiosis [1,2]. Compared to other cells in the organ, the MMC is distinguished by its large volume and prominent nucleus and nucleoli [3,4]. Although the formation of a single MMC is the general rule, multiple MMCs can occur in wild-type ovules, and MMC number varies among *Arabidopsis* ecotypes [5]. Mutants in diverse molecular pathways also display multiple MMCs: ectopic MMCs can be formed by neighboring subepidermal nucellar cells, or from ectopic divisions of a differentiated MMC [6]. Interestingly, these ectopic MMCs display a range of developmental outcomes. Ectopic MMCs can: (a) undergo normal progression to meiosis to generate a haploid germline [7,8], (b) skip meiosis to produce a non-reduced germline [9], and/or (c) abort reproductive differentiation and be re-incorporated in the soma [6,10]. These observations suggest that female reproductive commitment in the early ovule involves successive steps that allow both transient and stable reproductive fate acquisition.

Female reproductive fate acquisition occurs during ovule primordium growth, implying tight spatiotemporal coordination of cell divisions that multiply the soma or switch to the meiotic cell cycle to form the MMC. Inhibition of the core cell cycle machinery is required to stabilize meiotic commitment once the MMC is differentiated [7,8]. However, cell cycle regulation of *Arabidopsis* ovule primordia cells before MMC formation is not well understood. In some animal species, gamete precursors known as Primordial Germ Cells (PGCs), undergo proliferation followed by a cell cycle arrest phase before progression to germline formation [11,12]. Interestingly, while male PGCs arrest before the onset of meiotic DNA replication [13], female PGCs arrest at the leptotene stage of meiotic prophase I [11,14]. In *Arabidopsis*, exactly when female spore precursors enter the meiotic cell cycle is not known. However, DNA replication associated with meiotic S-phase has been observed in the MMC of ovules at stage 1-I [15]. We have also recently shown that subepidermal nucellar cells are mitotically quiescent at earlier stages of ovule emergence, and might form a pool of candidate MMCs [16]. Moreover, activation of S-phase-associated Proliferating Cell Nuclear Antigen (PCNA) is detected at least in one candidate MMC at early stages [16]. This suggests that the onset of meiotic S-phase could take place earlier in ovule development and is associated with mitotic arrest, as in animals. However, the question of whether reproductive fate acquisition is made prior, during, or after S-phase remains to be addressed.

Among the 15 *HISTONE THREE RELATED (HTR)* genes in *Arabidopsis*, five genes encode canonical histone 3.1 (H3.1) proteins, and three genes encode canonical H3.3 replacement histones [17]. In contrast to replication-independent H3.3 variants, most H3.1 histones are incorporated into chromatin of proliferating tissues during the S-phase of DNA replication, and maintained through the G2 and M phase progression [17,18,19]. Interestingly, in addition to its role in cell cycle progression, H3.1 turnover has been also related to cell cycle exit [19]. The fluorescent-tagged H3.1 variants HTR3 and HTR13 exhibited high and cycling reporter signal in the chromatin of proliferating cells, however their signals were absent in the quiescent center (QC) of embryos and the root apical meristem (RAM) of seedlings. H3.1 eviction was also observed in the last cell cycle of cells exiting the root meristem, in differentiated stomata guard cells, and at the end of the endocycle program in hypocotyls [19]. These data suggest that H3.1 eviction is characteristic of cell cycle exit events that result in either pluripotent stem cell fate or cell differentiation.

We reasoned that the study of H3.1 dynamics during MMC differentiation was key to understand pre-meiotic S-phase features and when mitotic cell cycle exit might occur during reproductive fate commitment in ovules. Using a fluorescent-tagged line, we found that the canonical H3.1 HTR13 is specifically evicted in the MMC, as described for QC cells in the embryo and the root. Surprisingly, at earlier stages of ovule primordia emergence, H3.1 eviction can occur in one, two or three subepidermal nucellar cells. This eviction persists at the MMC, even when S-phase occurs. Conversely, H3.1 is gradually re-incorporated in the chromatin of cells adjacent to the MMC. Our results suggest that cell cycle dynamics of H3.1 in the ovule primordium distinguish reproductive cells from somatic cells and mark cell fate plasticity during the somatic to reproductive transition. 

## 2. Results

To determine the pattern of H3.1 turnover during early ovule development [2], we used confocal microscopy to analyze *Arabidopsis* ovule primordia expressing an HTR13-GFP reporter encompassing the full *HTR13* genomic region [19]. Based on live-imaging experiments of HTR13-GFP in the root meristem, similar expression patterns observed using an independent HTR13-RFP line [19] and previous reports using an HTR13-CFP reporter [17], this HTR13-GFP reporter is a *bona fide* reporter of HTR13 protein dynamics.

**Figure 1:**
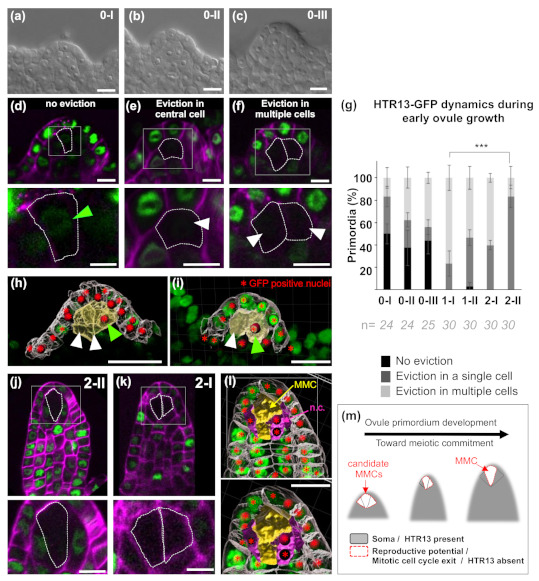
Specific dynamics of HTR13-GFP (H3.1) chromatin-deposition during early *Arabidopsis* ovule development. (**a**–**c**) Representative image of cleared ovules preceding stage 1-I (stages 0-I to 0-III), used for quantitative analysis. (**d**–**f**) Representative images of emerging ovule primordia showing the three different HTR13 patterns detected in subepidermal cells of the nucellus: (**d**): Ovule primordium showing no eviction of HTR13 at the MMC (Megaspore Mother Cell). Green arrow in the inset shows the nuclei of the MMC with GFP signal. (**e**) Ovule primordium showing HTR13 eviction (white arrow) in a single subepidermal cell. (**f**) Ovule primordium showing HTR13 eviction in two subepidermal cells (white arrows). (**g**) Frequency of ovule primordia exhibiting the distinct HTR13 patterns within subepidermal cells in the nucellus. Standard Error (SE) is indicated, n = number of ovules observed. Two-tailed Fischer’s test was used to compare the proportion of eviction in a single cell between stage 1-I and 2-II. *** *p* < 0.001. (**h**–**i**) 3D reconstructions of cell surfaces showing nuclei GFP signal detection (red stars) in all epidermal cells (grey surfaces), and only in one out of three (**h**) or in one out of two (**i**) subepidermal cells (yellow surfaces). (**j**) Ovule at stage 2-I showing eviction in the MMC and not in companion cells. (**k**) Ovule at stage 2-II showing eviction in two enlarged subepidermal cells. (**l**) 3D reconstruction of cell surfaces showing nuclear GFP signal in all epidermal cells, in MMC neighbor cells (magenta surfaces, n.c.) and eviction in the MMC (yellow surface). (**m**) Working model: At early stages of ovule primordium development, HTR13 eviction and cell cycle exit occur in several subepidermal cells, distinguishing them from the somatic tissues (soma). This eviction persists in the MMC, which will undergo meiosis. Conversely, HTR13 is gradually re-incorporated in the neighbor cells of the MMC, which re-enter the soma. Squares mark inset regions. Dashed lines mark MMC or candidate MMCs. Green: GFP fluorescence; Magenta: cell wall marker Renaissance SR2200; scale bars: 10 μm. (**a**–**c**) DIC microscopy. (**d**–**f**,**j**,**k**) Laser Scanning Confocal Microscopy. (**h**,**i**,**l**) 3D reconstruction of cell surfaces after epidermal and subepidermal cell segmentation and automated nuclei detection.

We investigated the dynamics of HTR13 in ovules at stages 1-I to 2-II [3]. We also examined three stages earlier than primordia stage 1-I, which have been recently named as 0-I, 0-II and 0-III [16] (Figure 1a–c). When examining subepidermal cells at the nucellar region, we could easily distinguish three different patterns: (a) HTR13-GFP signal in all subepidermal cells of the nucellus (Figure 1d); (b) HTR13-GFP signal in all but a single, central subepidermal cell of the nucellus (Figure 1e); (c) HTR13-GFP missing in multiple subepidermal cells of the nucellus (Figure 1f). Given the ubiquitous expression of HTR13-GFP in all cells of some ovules (Figure 1d), and the presence of *HTR13* mRNA in transcriptomes of the MMC and surrounding tissues [20], the absence of detectable HTR13-GFP signal in nucellar subepidermal cells was interpreted as post-transcriptional regulation, i.e., eviction of this tagged histone variant, similar to what has been described for H1 and H2A.Z histone variants in the MMC [15]. 

To determine the frequency and developmental timing of HTR13-GFP eviction in one or several subepidermal cells, we quantified the different patterns described above. At stage 0-I, 50% (n = 24) of ovules showed ubiquitous signal while 50% lacked a GFP signal at least in one subepidermal cell (Figure 1g). At the 0-II and 0-III stages, HTR13-GFP eviction was found in ~60% of ovules (Figure 1g, n = 24 and n = 25 for stages 0-II and 0-III, respectively). Strikingly, the occurrence of ovules where multiple subepidermal cells lack HTR13-GFP increased significantly at stage 0-III (2.6-fold in comparison with the frequency at stage 0-I) (Figure 1g). In contrast, epidermal cells consistently showed HTR13-GFP expression, as confirmed by scoring nuclei with GFP signal in the whole epidermal layer (99.1% ± 3%, n = 151 cells from seven ovules). Consistent signal was also observed in L3 and internal placenta layers, however no systematic quantifications were performed there. Such ubiquitous signal is distinct from the “salt and pepper” pattern characteristic of cell-cycle regulated genes observed for HTR13-GFP in the root meristem, but is similar to the pattern described in early embryos, where it was proposed to be related to a short G1 phase [19]. These observations suggest that H3.1 is specifically evicted in subepidermal cells at the apex of the ovule, starting early after ovule emergence, and might be related to their candidate MMC identity. This specific eviction may also be associated with reproductive competence of these cells in comparison to the rest of the cells in the organ, as suggested for H1 eviction occurring at stage 1-I [15].

To confirm that HTR13 eviction was associated with reproductive fate, we looked at later stage ovules displaying morphologically distinguishable MMCs. We found that up to 99.2% (n = 120) of primordia between stages 1-I and 2-II exhibited either a lack of fluorescent signal in the MMC (Figure 1j), or lack of signal in the MMC and also in one or two neighbor cells (Figure 1k). Only a residual number of primordia (3.3%, n = 30) presented a GFP signal in the MMC at stage 1-II (Figure 1g). Interestingly, the high proportion of eviction in multiple cells at stage 1-I (76.6%, n = 30) gradually decreased to 16.6% at stage 2-II so that 83.33% (n = 30) of ovules at this stage showed HTR13 eviction specifically within the MMC. This suggests that H3.1 eviction is linked to reproductive fate acquisition potential. The significant shift (Figure 1g, *p* < 0.001) between a large population of ovules showing H3.1 eviction in multiple cells, to a population of primordia mostly showing H3.1 eviction in a single MMC, suggests that neighboring cells reincorporate H3.1 into chromatin (Figure 1i–l). At stage 2-III, we observed HTR13 eviction in the MMC in 97% (n = 31) of ovules, while MMC neighbor cells expressed the marker (Figure S1).

Altogether, these results suggest that H3.1 eviction is a hallmark of candidate MMCs established at early stages of ovule primordium development. This eviction persists in the MMC, indicating that, in contrast to proliferative somatic cells, the H3.1 variant encoded by *HTR13* is not loaded into the chromatin during female meiotic S-phase. Conversely, neighbor cells of the MMC specifically reincorporate this histone variant at later stages. Thus, HTR13 dynamics also distinguish specific cell cycle features of MMC neighbor cells, as compared with other somatic cells. 

## 3. Discussion 

We found that multiple subepidermal cells of the nucellus displayed eviction of H3.1 from very early stages of ovule growth; and that this absence persists during pre-meiotic and meiotic stages [15,16]. This suggests that several cells in the nucellus are capable of engaging a program toward reproductive competence which can later progress to an MMC fate or a somatic fate. This is consistent with cellular markers like cell size, nuclear size and mitotic activity indicating that several candidate MMCs form in the ovule primordium, with a gradual canalization process that results in a single MMC [16]. The model supports the large and increasing genetic evidence demonstrating the capacity of several nucellar cells to acquire germline precursor identity [4,6,16,21]. 

Localized eviction of H3.1 in candidate MMCs in the nucellus, together with its persistence in other somatic cells within the ovule, is reminiscent of the pattern observed in *Arabidopsis* embryos at the heart and torpedo stages, where this same histone variant is depleted in the QC but maintained in other cells [19]. Such eviction is proposed to mark cell cycle exit toward pluripotency or differentiation, and has been observed in several cell types [19]. Eviction of H3.1 marking cell cycle exit in candidate MMCs is consistent with our recent observations that these cells are mitotically quiescent [16]. Interestingly, mitotic quiescence is detected in almost all primordia at stage 0-II [16], however HTR13 eviction appears only in a proportion of ovules at the same stage, suggesting that cell cycle progression at subepidermal cells within the nucellus is modified before HTR13 eviction. We also observed concomitant activation of the S-phase-associated PCNA pattern in a least one candidate MMC [16]. Hence our findings reinforce the hypothesis of an early transition to the meiotic S-phase during ovule primordium formation [16]. Based on these observations, we propose that H3.1 eviction within the ovule primordium marks the modification of cell cycle progression related to the acquisition of reproductive competence (Figure 1m). Whether such eviction is required to acquire MMC fate is a question for future investigation. 

Similar to the MMC, HTR13 is absent in mature *Arabidopsis* female and male germ cells [17,22], suggesting that HTR13 turnover is indeed necessary to establish germline identity. Limited global availability of H3.1 replicative histones might be associated with the specific cell cycle regulation of reproductive cells, as suggested for the egg cell where no or low signal for all H3.1 genes was detected [17]. Alternatively, the regulation observed in the MMC might be specific to HTR13 and potentially its related gene HTR3, which have been shown to display similar dynamics in roots and embryos [19]. Defining the MMC-specific turnover of the whole H3.1 subfamily should shed light on these alternatives. Active histone replacement mechanisms are expected for at least the H3.3 variants HTR5 and HTR8, which are present in the MMC [15]. In the root meristem, H3.1 eviction during the last cell cycle is associated with H3.3 replacement [19], and the H3.1/H3.3 ratio marks the proliferative status of the cells [23]. Consistent with this, during stages 1-II to 2-II, H3.1 is reloaded into the chromatin in MMC neighbor cells - which also contain H3.3 variants [15], increasing the H3.1/H3.3 ratio when these cells resume mitotic divisions [16]. Taken together, our observations that a pool of MMC candidates loses H3.1 and pauses their cell cycle [16] at stage 0-I, and at later stages, a single cell lacks H3.1 while the neighbors regain H3.1 and continue the mitotic cell cycle; indicate possible mechanisms contributing to the canalization process leading to a single MMC in the ovule. 

The precise link between cell cycle progression, chromatin reprogramming, and reproductive fate acquisition in plants remains largely unknown. In animals, differentiation of PGCs is accompanied by large-scale chromatin reprogramming, required for erasure of imprints, pluripotency establishment and meiotic entry [24,25]. Importantly, this reprogramming phase is concomitant with cell cycle exit, typically during G1, which is necessary for reproductive fate [12,26,27]. In the *Arabidopsis* ovule, in addition to extensive histone H3 variant remodeling within the MMC, depletion of the linker histones H1.1 and H1.2, followed by H2A.Z, takes place during stages 1-I to 2-I [15]. Interestingly, these reprogramming events are associated with the formation of ectopic MMCs in mutants such as *ago9* [15] and might contribute to the natural variation in MMC canalization observed in *Arabidopsis* [5], especially in ecotypes displaying decondensed chromatin states [28]. Histone turnover likely regulates marks required for cell cycle control and the switch to meiotic cycle [29], but could also reset somatic marks, as suggested for H3K27me3, which is significantly reduced in the MMC [15]. H3.1 eviction would be consistent with this possibility, since loss of function of the five H3.1 genes in *Arabidopsis* leads to a decrease of H3K27me3 in seedlings [30]. All these epigenetic mechanisms impinge on transcriptional landscape modifications. In germ cell precursors, they might favor the early expression of the meiotic transcriptional program, as shown in maize meiocytes [31].

Collectively, our results suggest a model where H3.1 eviction within candidate MMCs at the ovule apex during primordium emergence marks cell cycle exit and unlocks the potential of these cells to adopt reproductive competence. These findings shed light on the cellular and epigenetic mechanisms behind the striking developmental plasticity of the early ovule in angiosperms.

## 4. Material and Methods

### 4.1. Plant Materials

The *Arabidopsis* HTR13-GFP line is in the *Columbia* (Col-0) background and was previously reported in [19]. Plants were grown under long-day conditions (16 h light/8 h darkness) at 21–23 °C in a plant growth chamber. 

### 4.2. Microscopy and Image Analysis

For analysis of ovule primordia expressing the HTR13-GFP construct, we used gynoecia from floral buds at stages 8 to 12 [32], mounted in 5% glycerol with the cell wall stain Renaissance 2200 (SR2200) diluted 1:2000. Samples were observed using a Leica Laser Scanning Confocal Microscope LCS SP8, with a 63 × oil immersion lens (NA = 1.3). The GFP reporter was excited with a 488 nm wavelength and detected between 493 nm and 550 nm. The Renaissance fluorophore was excited with a 405 nm wavelength and detected between 415–476 nm. To obtain representative images of ovules preceding stage 1-I, we fixed floral buds at stage 8 in formalin-acetic acid-alcohol solution (40% formaldehyde, glacial acetic acid, 50% ethanol; in a 5: 5: 90 volume ratio) for 48 h at RT. Buds were then washed three times with absolute ethanol and placed in 70% ethanol for 24 h. Gynoecia were collected and placed in Herr’s clearing solution (phenol:chloral hydrate:85% lactic acid: xylene:clove oil, 1: 1: 1: 0.5: 1), and observed by differential interference contrast (DIC) microscopy using a Leica DMR microscope. Image contrast and intensity were adjusted using ImageJ/FIJI or OMERO.

### 4.3. Quantifications and Image Analysis.

Quantification of ovules presenting HTR13 eviction in zero, one or several subepidermal cells was performed by visual inspection in 3D through optical sectioning, directly under the confocal microscope. Quantification of HTR13 positive cells in the L1 layer was performed automatically on 3D stacks using IMARIS software (Bitplane, Zürich, Switzerland). Cell segmentation was done using cell wall Renaissance channel, by the procedure described in [33], and with the following parameters determined empirically: local contrast function with minimum cell diameter: 3µm; minimum cell wall thickness: 0.1 µm. The function “label” was used to manually identify L1 cells on 3D volume display. Manual curation of segmentation was performed, with the help of the GFP nuclei channel to validate ambiguous walls. Cells not correctly segmented were removed. Segmented L1 cells were exported as a “Surface” object, used to create a mask on the GFP nuclei channel. L1 GFP positive nuclei were scored within this masked surface using the spot detection function, and 2.5 µm as minimal nuclei diameter. Detection threshold was adjusted by ensuring that a single spot corresponded to each single nucleus. Using these parameters, the algorithm never detected nuclei in the differentiated MMC, providing a negative control. Total numbers of L1 cells and total numbers of GFP positive nuclei were extracted from IMARIS statistics. 

## Supplementary Materials

The following are available online at https://www.mdpi.com/2223-7747/9/10/1322/s1, Figure S1: HTR13-GFP pattern in ovules at stage 2-III.
