# Supplementary material for: H3.1 Eviction Marks Female Germline Precursors in Arabidopsis
Source: Plants (Basel). 2020 Oct 6;9(10):1322. doi: 10.3390/plants9101322 (PMC7600056; doi:10.3390/plants9101322)
Supplement: Supplementary file 1 [file plants-09-01322-s001.pdf]

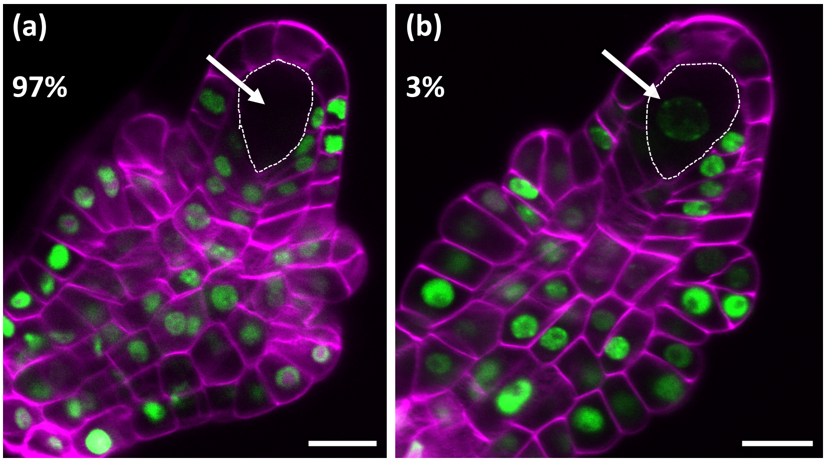

Figure S1. HTR13-GFP pattern in ovules at stage 2-III.

a: Representative image of stage 2-III ovule showing absence of GFP signal in the MMC (white arrow). This pattern was observed in 97% of the ovules (n=31). **b:** A weak nuclear signal in foci (white arrow) was observed in 3% of the ovules at stage 2-III. Dashed lines mark the MMC. Green: GFP fluorescence, Magenta: cell wall marker Renaissance SR2200, scale bar 10μm. **a-b:** Laser Scanning Confocal microscopy.
